# Role of Exosomes Released by Dendritic Cells and/or by Tumor Targets: Regulation of NK Cell Plasticity

**DOI:** 10.3389/fimmu.2014.00091

**Published:** 2014-03-07

**Authors:** Katrin S. Reiners, Juliane Dassler, Christoph Coch, Elke Pogge von Strandmann

**Affiliations:** ^1^Clinic I for Internal Medicine, University of Cologne, Cologne, Germany; ^2^Institute of Clinical Chemistry and Clinical Pharmacology, University Hospital Bonn, Bonn, Germany

**Keywords:** exosomes, NK cell regulation, tumor derived vesicles, dendritic cell-derived exosomes, microvesicles

## Abstract

Exosomes are endosomal-derived nanovesicles released by normal and tumor cells, which transfer functionally active proteins, lipids, and nucleic acids between cells. They are important mediators of intercellular communication and act on the adjacent stroma as well as in the periphery. Recently, exosomes have been recognized to play a pathophysiological role in various diseases such as cancer or infectious diseases. Tumor cell-derived exosomes (Tex) have been shown to act as tumor promotors by educating non-malignant cells to provide a tumor supporting microenvironment, which helps to circumvent immune detection by the host and supports metastasis. However, Tex with anti-tumor, immune-activating properties were also described reflecting the complexity of exosomes. Here, we assess the role of extracellular microvesicles/exosomes as messengers affecting NK cell function in health and disease and discuss the molecular basis for the differential impact of exosomes on NK cell activity. The molecular composition/load of exosomes and the mechanisms regulating their release remain unclear and need to be further analyzed to facilitate the development of new treatment options targeting the exosomal machinery.

## Introduction

Intercellular communication is a prerequisite for a functional immune surveillance in order to obtain a healthy organism. Virus infected or malignant cells are identified and eliminated by the immune system via membrane bound or secreted molecules.

Besides these well-known pathways of cell–cell communication the impact of endosomal-derived microvesicles, so called exosomes, has emerged as another important mechanism to regulate the immune response.

The function of exosomes was originally attributed to the elimination of obsolete proteins, however, it became quite clear that these vesicles are important mediators of intercellular communication. Exosomes are a homogeneous group of nanovesicles with a size of approximately 50–100 nm. They are budded into the limiting membrane of endosomes to form multivesicular bodies (MVB). The fusion of the MVB with the plasma membrane leads to the release of the intraluminal vesicles into the extracellular space.

Exosomes are specifically loaded with membrane and cytosolic proteins as well as nucleic acids, depending on the originating cell and its homeostatic state. This results in the formation of functionally distinct exosomes and their activity ranges from immune activation to immune suppression or induction of tolerance. However, the mechanisms that regulate the exosomal release and the sorting of specific molecules in exosomes are far from being understood. In this perspective, we focus on the interaction of exosomes with NK cells.

## Dendritic Cell-Derived Exosomes: Potent Activators of NK Cells

The ability to activate NK cells has been primarily reported for exosomes derived from dendritic cells (DCs). Dendritic cell-derived exosomes (Dex) can directly trigger NK cell activation in mice and cancer patients. The interaction of TNF expressed on Dex with the corresponding TNF receptors on NK cells induced interferon-γ (IFNγ) secretion from NK cells. These data demonstrate that Dex can mediate essential innate immune functions – a feature that was previously ascribed to DCs themselves (1). The use of Dex in clinical phase I trials was reported to trigger NK cells in half of the patients (2–4), but the underlying mechanisms for the Dex bioactivity on NK cells remained unclear. In 2009, Viaud et al. showed that membrane bound ligands for NKG2D as well as IL-15Ra seemed to play an essential role in Dex-induced activation of NK cells in humans and mice (5). Thereby, Dex triggered an IL-15Ra and NKG2D-dependent NK cell proliferation and activation in secondary lymphoid organs in mice. A first phase I clinical study with 15 melanoma patients supported the notion that Dex vaccines significantly enhanced the number of circulating NK cells and restored their NKG2D expression levels (5). Furthermore, exosomes released from DCs expressed BAG6, a ligand for the activating NK cell receptor NKp30. BAG6-expressing exosomes induced NK cell-mediated cytokine release and cytotoxicity, indicating a role for exosomes released from DCs to initiate the innate function of NK cells (6). The exosomal release of danger signals that alert NK cells may be considered as priming signal and supports a two-step activation model of human NK cells involving a priming and a triggering event.

As Dex also express functional MHC molecules and T cell co-stimulatory molecules such as CD40, CD80, and CD86 (4), they are also potent T cell activators (reviewed elsewhere). In addition, Dex can directly mediate tumor cell killing via Fas-L and TRAIL (1). This combination of direct anti-tumor and immune stimulatory activity seem to make Dex ideal tools for cancer treatment. Nevertheless, Dex therapies were so far not expanded into the clinical routine. This might be attributed to technical difficulties to produce sufficient amounts of exosomes with defined properties. A better basic understanding of the regulation, biosynthesis, and specific protein and RNA cargo of exosomes from different cell types and environments would definitely inspire the development of novel therapies, which might in the end be based on *in vivo* modulation of exosome release and function. This holds also true for exosomes released from tumor cells (Tex). Tumor-derived exosomes are predominantly described as immune-suppressing vesicles, however, there are also reports of anti-tumor immune-activating Tex. As soon as we understand the signals directing the formation of functional distinct exosomes, we can proceed and modify the exosomes and their release therapeutically to retain their anti-tumor activity.

## Tumor Cell-Derived Exosomes: Benefit or Danger?

Tumor cells develop a number of mechanisms to escape or suppress an active immune response such as down-regulation of surface MHC molecule expression (7), secretion of immune-inhibitory cytokines (8) or by regulating stromal components to generate a tumor growth promoting microenvironment (9).

More recently, the impact of tumor cell-derived exosomes on immune surveillance has been discussed. While the effect of tumor cell-derived exosomes (Tex) on T cells is extensively investigated, little is published on the direct impact of Tex on NK cell function. Unlike for Dex, Tex are discussed to be immune-activating as well as immune-inhibitory, although reports on Tex with immune-stimulating function are clearly outnumbered by studies indicating an inhibitory effect on the immune response.

### Tex as immune stimulators

As Tex have the ability to express tumor-associated antigens, they play a role in cancer immunology, such as transport of antigens to DCs to initiate an anti-tumor immune response via cross-presentation (10, 11). HepG2 and PLC/PRF/5 cell lines were used as models to study heat shock protein (Hsp)-bearing exosome secretion by hepatocellular carcinoma cells under stress conditions (12). Their results showed that incubation of NK cells with Hsp-bearing exosomes augmented cytolytic activity against K562 or HepG2 target cells through granzyme B release; up-regulation of activating receptors CD69, NKG2D, and NKp44; and down-regulation of inhibitory receptor CD94. This seemed to be dependent not only on exosome concentration but also on Hsp expression, with notably higher Hsp expression on HepG2-released exosomes after treatment with chemotherapeutics. Interestingly, treatment with resistant anti-cancer drugs seemed to enhance Hsp expression on exosomes more efficiently than sensitive chemotherapeutics, leading to a more pronounced NK cell activation (12). This is in line with findings of Gastpar and colleagues, who showed that NK cells were stimulated by human pancreas and colon carcinoma sublines-derived exosomes, depending on their capacity to present heat shock protein 70 (Hsp70)/Bag-4 on their membranes. Natural killer cells were stimulated selectively by Hsp70/Bag-4 surface-positive exosomes; an effect that could be blocked with Hsp70-blocking antibody (13). BAG6, another Hsp70-interacting protein, is known to engage the activating NK cell receptor NKp30 and BAG6-expressing exosomes trigger NK cell-mediated cytokine release and cytotoxicity (14, 15). Moreover, Tex may induce the up-regulation of granzyme B, IL-2, IFNγ, TNFα, CD25, and reduce CD95L expression in NK cells (16), further arguing for Tex-supported NK cell activation.

Still, Tex are mostly described as inhibitors of the immune system supporting tumor immune evasion, indicating that the formation of NK cell-activating exosomes may depend on the tumor cell type, state of tumor progression, and the microenvironment, factors that still need to be defined.

### Tex as immune inhibitors

Several reports state an immune suppressive effect of tumor cell-derived exosomes on NK cells. This effect is frequently associated with an altered surface protein expression. Often, ligands for the activating NK cell receptor NKG2D are being identified as crucial factors.

Clayton et al. demonstrated that NKG2D is down regulated on CD3-positive peripheral blood leukocytes following exposure to tumor-derived exosomes, resulting in an impaired cytotoxic effector function of the CD8^+^ T cells (17). This effect was dependent on the exosomal expression of NKG2D ligands (NKG2D-L) and could be abolished by blocking with corresponding NKG2D ligand antibodies. Yet, in this study the effects of Tex were only detected on T cells, but not proven for CD3-negative PBL. Subsequent publications indicate that NKG2D-L-bearing exosomes can have similar effects on NK cells. For instance, incubation of NK cells with MICA-containing exosomes provokes an NKG2D-dependent reduction of NK cell cytotoxicity independent of NKG2D-L expression by the target cell (18). In a further study, NKG2D-L-expressing tumor-derived exosomes showed a direct interaction with NK cells and CD8^+^ T cells leading to a significant reduction in cell surface NKG2D expression (19). This was at least partly dependent on NKG2D-L; however, in this study the impact of exosomal TGFbeta1 on the observed effects was much stronger. Exosome-mediated down-modulation of NKG2D correlated with poor functional responses, as the interaction resulted in an impaired cytolytic function of NK cells. Thus, the efficiency of Tex might reflect their specific feature to display multiple inhibitory signals at once and by this being able to even overcome pro-inflammatory signals. This is in line with findings reporting an inhibition of IL-2-mediated up-regulation of CD25 by tumor exosomes selectively in NK cells and CD8^+^ T cells (20). At the same time, CD4^+^CD25^+^ Treg cells remained IL-2 responsive and their inhibitory function was enhanced by tumor exosomes. Liu and colleagues showed that exosomes derived from murine mammary carcinoma cell lines inhibit NK cell cytotoxic activity *ex vivo* and *in vitro*. Key features of NK cell activity were inhibited, including release of perforin but not granzyme B, as well as the expression of cyclin D3 and activation of Jak3-mediated pathways. Human tumor cell lines were also found to produce exosomes that were capable of inhibiting IL-2-stimulated NK cell proliferation. Thus, it was proposed that tumor exosomes contribute to the growth of tumors by blocking IL-2-mediated activation of NK cells and their cytotoxic response to tumor cells (21).

Clinical observations revealed that sera of acute myeloid leukemia (AML) patients have higher levels of microvesicles with an explicit molecular profile expressing membrane-associated TGFbeta1, MICA/MICB, and the myeloid blast markers CD34, CD33, CD117 (22). Again, TGFbeta1 seemed the prominent factor to induce immune suppression by decreasing NK cell cytotoxicity and down-regulation of NKG2D expression. In this setting, treatment with IL-15 protected NK cells from adverse effects of tumor-derived microvesicles. Findings on exosomes/microvesicles isolated from solid tumors or AML patient sera and their modulatory functions on different immune cells were recently summarized and comprehensively reviewed (23). In gastric cancer patients, elevated Fas levels have been detected on tumor-infiltrating as well as circulating NK cells. This was closely related to a significantly increased NK cell apoptosis (24). Thus, exosomes expressing death receptor ligands might not only be able to kill T cells (25–27) but it seems quite likely that they will have similar effects on NK cells.

Taken together, via diverse mechanisms acting in concert, Tex potentially contribute to an immunosuppressive environment, emphasizing their physiological importance for tumor immune escape. The activity of NKG2D-L on Tex is obviously contradictory to the NKG2D-L activity on Dex, which are known to activate NK cells. This reflects the context-dependent impact of a given molecule on exosomes – which further strengthens the need for a better basic understanding of exosome regulation and formation.

### Exosome cargo formation

In summary, the composition of exosomes with proteins and nucleic acids seems to be decisive regarding their ability to inhibit or promote an immune response. Most likely, the sorting is not solely modulated by the transported protein itself, as NKG2D-L are nicely targeted to exosomes in the breast cancer cell line T47d, whereas exosomes of the strongly NKG2D-L positive T cell lymphoma line Jurkat are completely NKG2D-L negative (17). Ashiru et al. stated a putative sorting mechanism for the NKG2D-L MICA. They speculate that the specific translocation of MICA*008 into exosomes is in contrast to other MICA/B molecules due to its difference in the predicted transmembrane and cytoplasmic domains (18). Recently, the group was able to demonstrate that GPI modification of MICA*008 is responsible for the recruitment into exosomes (28). Nevertheless, today it is still little known about the mechanisms of protein sorting into exosomes. Some exosomal proteins are sorted by the ubiquitin pathway (29, 30). Current findings suggest that tetraspanin-enriched microdomains may act as compartmentalizing platforms that aid the selection of some intracellular components toward exosomes, as genetic deletion of the tetraspanin CD81 impaired the inclusion of a number of selective CD81-binding proteins into exosomes (31). Furthermore, the contribution of the ESCRT (endosomal sorting complex required for transport)-machinery is discussed (32–34). It was also stated that the formation of proteolipid protein-containing exosomes does not require the ESCRT machinery, but sphingolipid ceramide and the activity of the neutral sphingomyelinase (35). Proteins expressed on exosomes do not share a general exosome-targeting amino acid motif, but are rather characterized by a broad range of sequence motifs that confer plasma membrane binding and higher-order oligomerization (36). This might be different for the specific loading of RNA species into exosomes. Recently it was reported that A2B1 (heterogeneous nuclear ribonucleoprotein hnRNPA2B1) specifically recognizes and binds sequence motifs present in miRNAs to control their packaging into exosomes (37).

**Figure 1:**
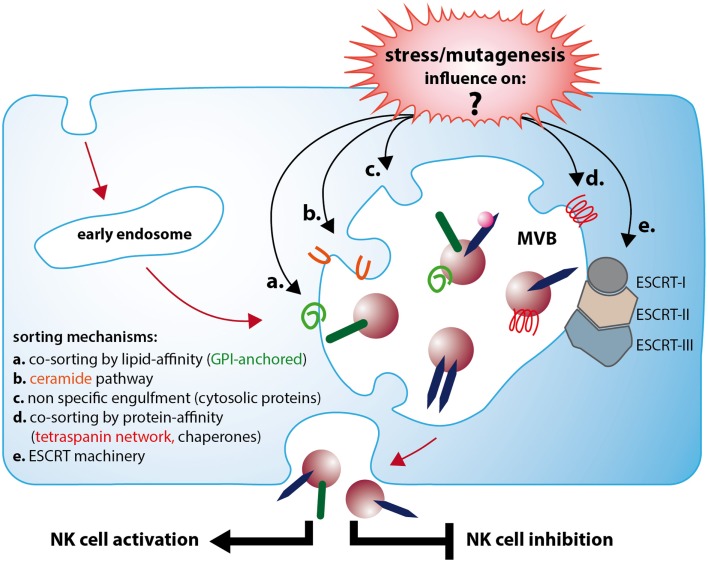
**Exosomes released from stressed or neoplastic cells can lead to NK cell activation or inhibition, which depends on a differential molecular composition**. Immune-activating exosomes (e.g., Dex) often express TNF and can induce INFγ secretion and enhance cytolytic activity of NK cells. In contrast, immune suppressive exosomes often contain TGFbeta1 and NKG2D ligands and inhibit NK cell cytotoxicity. Cellular stress and mutagenesis affect the biogenesis of functional distinct exosomes. Although several features attributed to one or the other effect are described, the mechanisms leading to the respective exosome formation are still poorly understood. A major question remaining to be answered is how stress and mutagenesis affect the different sorting mechanisms responsible for the genesis of immune-activating or -suppressive vesicles. As soon as the determining pathways and their regulation are comprehensively explored, the exosome cargo will become more predictable and allow the development of new, astute strategies to employ exosomes as therapeutic tools.

However, the signaling pathways that direct the specific loading of proteins and nucleic acids of different cell types in response to the cell status and environment remain largely unknown (see Figure 1). Recently, it was shown that viral stress signals may alter the composition of exosomes and by this can change their immune-modulatory capacity. The activation of the cytosolic pattern recognition receptor (PRR) RIG-I with its respective ligand leads to an enhanced release of NK cell-activating exosomes from melanoma cells. Thus, former tumor-promoting exosomes were transformed to immune-activating, anti-tumor immune agents. This pathway establishes a novel and unexpected link of a PRR and the release of exosomes (Dassler and Reiners, unpublished work). In this context, the impact of TLR activation on exosome release and characteristics seems an interesting topic that deserves to be studied in more detail.

## Conclusion

The multiple functions of exosomes make them somewhat double edged features for new therapeutic strategies: on the one hand, they appear to be especially suitable, as they may be selectively loaded with optimal drug combinations, have an excellent biodistribution and biocompatibility and can act with different mechanisms at once. On the other hand, to employ exosomes as anti-tumor reagent, a better understanding on exosomes target selection is required to improve therapeutic exosome application. Exosomes are suggested to bind to and be taken-up by selected targets (18). For instance, exosomes are rich in tetraspanins and some studies suggest exosomal tetraspanin–integrin complexes to be involved in target cell binding (38). Rana et al. were able to show that even minor differences in exosomal tetraspanin-complexes strongly influence target cell selection *in vitro* and *in vivo*. Their report on the contribution of exosomal tetraspanins to target cell selection is one step toward the ability to predict potential target cells.

As long as we do not thoroughly understand the mechanisms of protein (and nucleic acid) sorting into exosomes, the outcome of any new therapeutic strategy involving exosomes will be sparsely predictable. Only the basic knowledge on how the molecular composition of exosomes is determined and how exosome biogenesis is influenced by cell status, the environment or signaling will enable us to develop new, innovative strategies with exosomes as tools or targets.

## Conflict of Interest Statement

The authors declare that the research was conducted in the absence of any commercial or financial relationships that could be construed as a potential conflict of interest.

## References

[1] Munich S Sobo-Vujanovic A Buchser WJ Beer-Stolz D Vujanovic NL Dendritic cell exosomes directly kill tumor cells and activate natural killer cells via TNF superfamily ligands. Oncoimmunology (2012) 1(7):1074–83 10.4161/onci.20897 23170255 PMC3494621

[2] Escudier B Dorval T Chaput N Andre F Caby MP Novault S Vaccination of metastatic melanoma patients with autologous dendritic cell (DC) derived-exosomes: results of the first phase I clinical trial. J Transl Med (2005) 3(1):10 10.1186/1479-5876-3-10 15740633 PMC554765

[3] Morse MA Garst J Osada T Khan S Hobeika A Clay TM A phase I study of dexosome immunotherapy in patients with advanced non-small cell lung cancer. J Transl Med (2005) 3(1):9 10.1186/1479-5876-3-9 15723705 PMC551593

[4] Chaput N Flament C Viaud S Taieb J Roux S Spatz A Dendritic cell derived-exosomes: biology and clinical implementations. J Leukoc Biol (2006) 80(3):471–8 10.1189/jlb.0206094 16809645

[5] Viaud S Terme M Flament C Taieb J Andre F Novault S Dendritic cell-derived exosomes promote natural killer cell activation and proliferation: a role for NKG2D ligands and IL-15Ralpha. PLoS One (2009) 4(3):e4942 10.1371/journal.pone.0004942 19319200 PMC2657211

[6] Simhadri VR Reiners KS Hansen HP Topolar D Simhadri VL Nohroudi K Dendritic cells release HLA-B-associated transcript-3 positive exosomes to regulate natural killer function. PLoS One (2008) 3(10):e3377 10.1371/journal.pone.0003377 18852879 PMC2566590

[7] Doubrovina ES Doubrovin MM Vider E Sisson RB O’Reilly RJ Dupont B Evasion from NK cell immunity by MHC class I chain-related molecules expressing colon adenocarcinoma. J Immunol (2003) 171(12):6891–9 14662896 10.4049/jimmunol.171.12.6891

[8] Beck C Schreiber H Rowley D Role of TGF-beta in immune-evasion of cancer. Microsc Res Tech (2001) 52(4):387–95 10.1002/1097-0029(20010215)52:4<387::AID-JEMT1023>3.0.CO;2-W 11170297

[9] Gajewski TF Schreiber H Fu YX Innate and adaptive immune cells in the tumor microenvironment. Nat Immunol (2013) 14(10):1014–22 10.1038/ni.2703 24048123 PMC4118725

[10] Wolfers J Lozier A Raposo G Regnault A Thery C Masurier C Tumor-derived exosomes are a source of shared tumor rejection antigens for CTL cross-priming. Nat Med (2001) 7(3):297–303 10.1038/85438 11231627

[11] Andre F Schartz NE Movassagh M Flament C Pautier P Morice P Malignant effusions and immunogenic tumour-derived exosomes. Lancet (2002) 360(9329):295–305 10.1016/S0140-6736(02)09552-1 12147373

[12] Lv LH Wan YL Lin Y Zhang W Yang M Li GL Anticancer drugs cause release of exosomes with heat shock proteins from human hepatocellular carcinoma cells that elicit effective natural killer cell antitumor responses in vitro. J Biol Chem (2012) 287(19):15874–85 10.1074/jbc.M112.340588 22396543 PMC3346092

[13] Gastpar R Gehrmann M Bausero MA Asea A Gross C Schroeder JA Heat shock protein 70 surface-positive tumor exosomes stimulate migratory and cytolytic activity of natural killer cells. Cancer Res (2005) 65(12):5238–47 10.1158/0008-5472.CAN-04-3804 15958569 PMC1785299

[14] Pogge von Strandmann E Simhadri VR von Tresckow B Sasse S Reiners KS Hansen HP Human leukocyte antigen-B-associated transcript 3 is released from tumor cells and engages the NKp30 receptor on natural killer cells. Immunity (2007) 27(6):965–74 10.1016/j.immuni.2007.10.010 18055229

[15] Reiners KS Topolar D Henke A Simhadri VR Kessler J Sauer M Soluble ligands for NK cell receptors promote evasion of chronic lymphocytic leukemia cells from NK cell anti-tumor activity. Blood (2013) 121(18):3658–65 10.1182/blood-2013-01-476606 23509156 PMC3643764

[16] Zech D Rana S Buchler MW Zoller M Tumor-exosomes and leukocyte activation: an ambivalent crosstalk. Cell Commun Signal (2012) 10(1):37 10.1186/1478-811X-10-37 23190502 PMC3519567

[17] Clayton A Tabi Z Exosomes and the MICA-NKG2D system in cancer. Blood Cells Mol Dis (2005) 34(3):206–13 10.1016/j.bcmd.2005.03.003 15885603

[18] Ashiru O Boutet P Fernandez-Messina L Aguera-Gonzalez S Skepper JN Vales-Gomez M Natural killer cell cytotoxicity is suppressed by exposure to the human NKG2D ligand MICA*008 that is shed by tumor cells in exosomes. Cancer Res (2010) 70(2):481–9 10.1158/0008-5472.CAN-09-1688 20068167 PMC2817492

[19] Clayton A Mitchell JP Court J Linnane S Mason MD Tabi Z Human tumor-derived exosomes down-modulate NKG2D expression. J Immunol (2008) 180(11):7249–58 18490724 10.4049/jimmunol.180.11.7249

[20] Clayton A Mitchell JP Court J Mason MD Tabi Z Human tumor-derived exosomes selectively impair lymphocyte responses to interleukin-2. Cancer Res (2007) 67(15):7458–66 10.1158/0008-5472.CAN-06-3456 17671216

[21] Liu C Yu S Zinn K Wang J Zhang L Jia Y Murine mammary carcinoma exosomes promote tumor growth by suppression of NK cell function. J Immunol (2006) 176(3):1375–85 16424164 10.4049/jimmunol.176.3.1375

[22] Szczepanski MJ Szajnik M Welsh A Whiteside TL Boyiadzis M Blast-derived microvesicles in sera from patients with acute myeloid leukemia suppress natural killer cell function via membrane-associated transforming growth factor-beta1. Haematologica (2011) 96(9):1302–9 10.3324/haematol.2010.039743 21606166 PMC3166100

[23] Whiteside TL Immune modulation of T-cell and NK (natural killer) cell activities by TEXs (tumour-derived exosomes). Biochem Soc Trans (2013) 41(1):245–51 10.1042/BST20120265 23356291 PMC3721347

[24] Saito H Takaya S Osaki T Ikeguchi M Increased apoptosis and elevated Fas expression in circulating natural killer cells in gastric cancer patients. Gastric Cancer (2013) 16(4):473–9 10.1007/s10120-012-0210-1 23179366

[25] Andreola G Rivoltini L Castelli C Huber V Perego P Deho P Induction of lymphocyte apoptosis by tumor cell secretion of FasL-bearing microvesicles. J Exp Med (2002) 195(10):1303–16 10.1084/jem.20011624 12021310 PMC2193755

[26] Huber V Fais S Iero M Lugini L Canese P Squarcina P Human colorectal cancer cells induce T-cell death through release of proapoptotic microvesicles: role in immune escape. Gastroenterology (2005) 128(7):1796–804 10.1053/j.gastro.2005.03.045 15940614

[27] Kim JW Wieckowski E Taylor DD Reichert TE Watkins S Whiteside TL Fas ligand-positive membranous vesicles isolated from sera of patients with oral cancer induce apoptosis of activated T lymphocytes. Clin Cancer Res (2005) 11(3):1010–20 10.1158/1078-0432.CCR-04-1908 15709166

[28] Ashiru O Lopez-Cobo S Fernandez-Messina L Pontes-Quero S Pandolfi R Reyburn HT A GPI anchor explains the unique biological features of the common NKG2D-ligand allele MICA*008. Biochem J (2013) 454(2):295–302 10.1042/BJ20130194 23772752

[29] Buschow SI Liefhebber JM Wubbolts R Stoorvogel W Exosomes contain ubiquitinated proteins. Blood Cells Mol Dis (2005) 35(3):398–403 10.1016/j.bcmd.2005.08.005 16203162

[30] Zhang HG Kim H Liu C Yu S Wang J Grizzle WE Curcumin reverses breast tumor exosomes mediated immune suppression of NK cell tumor cytotoxicity. Biochim Biophys Acta (2007) 1773(7):1116–23 10.1016/j.bbamcr.2007.04.015 17555831 PMC2577190

[31] Perez-Hernandez D Gutierrez-Vazquez C Jorge I Lopez-Martin S Ursa A Sanchez-Madrid F The intracellular interactome of tetraspanin-enriched microdomains reveals their function as sorting machineries toward exosomes. J Biol Chem (2013) 288(17):11649–61 10.1074/jbc.M112.445304 23463506 PMC3636856

[32] Baietti MF Zhang Z Mortier E Melchior A Degeest G Geeraerts A Syndecan-syntenin-ALIX regulates the biogenesis of exosomes. Nat Cell Biol (2012) 14(7):677–85 10.1038/ncb2502 22660413

[33] Hurley JH Odorizzi G Get on the exosome bus with ALIX. Nat Cell Biol (2012) 14(7):654–5 10.1038/ncb2530 22743708

[34] Colombo M Moita C van Niel G Kowal J Vigneron J Benaroch P Analysis of ESCRT functions in exosome biogenesis, composition and secretion highlights the heterogeneity of extracellular vesicles. J Cell Sci (2013) 126(Pt 24):5553–65 10.1242/jcs.128868 24105262

[35] Trajkovic K Hsu C Chiantia S Rajendran L Wenzel D Wieland F Ceramide triggers budding of exosome vesicles into multivesicular endosomes. Science (2008) 319(5867):1244–7 10.1126/science.1153124 18309083

[36] Yang JM Gould SJ The cis-acting signals that target proteins to exosomes and microvesicles. Biochem Soc Trans (2013) 41(1):277–82 10.1042/BST20120275 23356297

[37] Villarroya-Beltri C Gutierrez-Vazquez C Sanchez-Cabo F Perez-Hernandez D Vazquez J Martin-Cofreces N Sumoylated hnRNPA2B1 controls the sorting of miRNAs into exosomes through binding to specific motifs. Nat Commun (2013) 4:2980 10.1038/ncomms3980 24356509 PMC3905700

[38] Rana S Yue S Stadel D Zoller M Toward tailored exosomes: the exosomal tetraspanin web contributes to target cell selection. Int J Biochem Cell Biol (2012) 44(9):1574–84 10.1016/j.biocel.2012.06.018 22728313

